# Effects of Barbell Deadlift Training on Submaximal Motor Unit Firing Rates for the Vastus Lateralis and Rectus Femoris

**DOI:** 10.1371/journal.pone.0115567

**Published:** 2014-12-22

**Authors:** Matt S. Stock, Brennan J. Thompson

**Affiliations:** Muscular Assessment Laboratory, Texas Tech University, Lubbock, TX, United States of America; The University of Queensland, Australia

## Abstract

Previous investigations that have studied motor unit firing rates following strength training have been limited to small muscles, isometric training, or interventions involving exercise machines. We examined the effects of ten weeks of supervised barbell deadlift training on motor unit firing rates for the vastus lateralis and rectus femoris during a 50% maximum voluntary contraction (MVC) assessment. Twenty-four previously untrained men (mean age  = 24 years) were randomly assigned to training (*n* = 15) or control (*n* = 9) groups. Before and following the intervention, the subjects performed isometric testing of the right knee extensors while bipolar surface electromyographic signals were detected from the two muscles. The signals were decomposed into their constituent motor unit action potential trains, and motor units that demonstrated accuracy levels less than 92.0% were not considered for analysis. One thousand eight hundred ninety-two and 2,013 motor units were examined for the vastus lateralis and rectus femoris, respectively. Regression analyses were used to determine the linear slope coefficients (pulses per second [pps]/% MVC) and y-intercepts (pps) of the mean firing rate and firing rate at recruitment versus recruitment threshold relationships. Deadlift training significantly improved knee extensor MVC force (Cohen's *d* = .70), but did not influence force steadiness. Training had no influence on the slopes and y-intercepts for the mean firing rate and firing rate at recruitment versus recruitment threshold relationships. In agreement with previous cross-sectional comparisons and randomized control trials, our findings do not support the notion that strength training affects the submaximal control of motor units.

## Introduction

Exercise physiologists have had an interest in the adaptations that occur as a result of strength training for decades. While the changes in muscle size and the transformation from type IIx to IIa fibers associated with heavy strength training are well documented [1], [2], comparatively less is known about the adaptations of individual motor units during voluntary contractions, particularly for those with high recruitment thresholds [3]. Within the last 15 years, a number of studies have examined the effects of strength training on the firing rates of motor units [4]–[10]. The ability to form a general consensus on the results of these studies is difficult, however, because a variety of methodological approaches and time courses have been used. Patten et al. [7] examined maximal firing rates of the abductor digiti minimi following strength training in young versus elderly adults. It was reported that maximal firing rates were 24% greater 48 hours following the pre-test, and the magnitude of increase was more pronounced for the elderly (29.5%) versus the young adults (18.2%). Interestingly, the firing rates decreased thereafter, and by six weeks into the training program, they had nearly returned to baseline levels. In contrast to adaptations demonstrated for maximal firing rates, authors that have studied submaximal contractions have not consistently demonstrated changes as a result of training. Vila-Cha et al. [9] used intramuscular electromyography (EMG) to assess submaximal firing rates for the vastus lateralis and vastus medialis following six weeks of strength training versus endurance exercise. Opposite results were demonstrated for the two modes of exercise, with small increases and decreases in firing rates during a 30% maximum voluntary contraction (MVC) assessment for strength and endurance training, respectively. In addition, investigators that have used signal processing techniques that allow for the analysis of variables other than maximal and/or mean firing rates have not observed effects associated with strength training [6], [11]. Kidgell et al. [6] assessed motor unit synchronization and coherence analyses to conclude that strength training of the hand did not induce neuromuscular adaptations for the first dorsal interosseous. Using cross-sectional comparisons, De Luca et al. [11] found no difference in the common drive of motor units among control subjects, skilled musicians, swimmers, and competitive powerlifters.

The ability to non-invasively examine the firing rates of motor units via surface EMG signal decomposition has recently been described in the literature [12]–[18]. As described by Nawab et al. [17], improvements in signal processing have resolved many of the complex challenges associated with an accurate decomposition. These challenges include action potentials superimposed on each other, shape changes throughout a contraction, and a large dynamic range. Most notably, updates to the Precision Decomposition algorithm allow for the analysis of significantly more motor units than what has previously been described in motor control studies. This increase can largely be explained by both improvements in the decomposition algorithm and differences in pickup area for intramuscular versus surface EMG signals [12], [17]. The use of this technology allows researchers to noninvasively quantify the recruitment, derecruitment, and firing statistics (e.g., synchronization, mean firing rates, common drive) of motor units, which may be valuable for answering a variety of research questions. By combining motor unit data with regression-based statistical analyses, Beck et al. [4] examined the effects of strength training on the linear slope coefficient of the mean firing rate versus recruitment threshold relationship. The y-intercept of the relationship was not examined. These authors [4] hypothesized that eight weeks of strength training would systemically increase the firing rates of the high threshold vastus lateralis motor units, thereby causing a decrease in the linear slope coefficient of this relationship via equivalency of all mean firing rates (i.e., slope of zero). In contrast to their research hypothesis, Beck et al. [4] concluded that the training program did not affect this relationship. It is important to note, however, that an increase in the group mean MVC value was reported, but only relative force levels (80% MVC) were studied. In addition to mean firing rates during the constant-force portion of a contraction, the analysis of motor unit behavior may also include the firing rates upon recruitment [13]. Like the association between the mean firing rates of motor units and their respective recruitment thresholds, the relationship between firing rate at recruitment and recruitment threshold also appears to be negative [13]. While this dependent variable has been studied for the vastus lateralis, no previous researchers have examined the rectus femoris. It is also unclear if this relationship may be influenced by chronic strength training.

Authors that have studied the effects of strength training on motor unit firing rates have studied small muscles of the hand [6], [7], maximal isometric exercise [8], or had subjects use exercise machines [4], [5], [9]. These studies have been very beneficial for answering important research questions concerning the control of motor units. However, the ability to generalize such findings to a wide audience is somewhat limited, since exercise interventions involving small muscles of the hand or maximal isometric contractions are typically not recommended aspects of exercise programs in healthy populations. Rather, experts generally agree and recommend that large muscle mass exercises that stress multiple joints be emphasized within the context of a training program designed to enhance muscular strength and performance [19], [20]. The present study was undertaken to examine changes in the mean firing rates of motor units, as well as the firing rates at recruitment, for both the vastus lateralis and rectus femoris following ten weeks of supervised deadlift training. The deadlift is an exercise that involves extension at the knee and hip joints, and relies heavily on force production from the vastus lateralis and rectus femoris [21]. This investigation differs from previous methodological approaches [4], [5] due to the fact that we examined two force levels during post-testing: 1) 50% of the pre-test MVC and 2) 50% of the post-test MVC. In agreement with the results reported by Beck et al. [4], it was our hypothesis that when the same relative force level was examined for both the pre- and post-test, there would be no statistical changes in the linear slope coefficients and y-intercepts for the mean firing rate versus recruitment threshold relationships for the two muscles. In contrast, it was our belief that for subjects demonstrating a large and meaningful increase in knee extensor MVC force, the slopes and y-intercepts would be affected during the absolute force measurements. In particular, we hypothesized that the y-intercept values would be lower due to lower firing rates, which is consistent with the fact that less motor unit activity is required to produce a given absolute, submaximal force following progressive strength training [22]. We further hypothesized that: 1) the changes demonstrated following training for the mean firing rate statistics would mirror those for the firing rate at recruitment and 2) the firing rate statistics for the vastus lateralis and rectus femoris would be comparable due to their common innervation from the femoral nerve and similar fiber type composition [23].

## Methods

### Subjects

Twenty-six men volunteered to participate in this study. Upon enrollment, the subjects were randomly assigned to strength training (*n* = 15) and control (*n* = 11) groups. Two subjects in the control group had to be removed from the dataset due to the accuracy and decomposition requirements described below. Thus, data has been presented for twenty-four men (mean ± SD age  = 24±3 years; body mass  = 83.0±17.4 kg). All subjects were healthy and not affected by neuromuscular and/or musculoskeletal disorders. Each subject had refrained from lower-body strength training during at least the previous six months. This investigation and its methods were approved by the Texas Tech University Human Research Protection Program. The project's approval number was 504943. All subjects read, understood, and signed an informed consent form, and completed a health history questionnaire prior to participation. The subjects in the control group were asked to refrain from lower-body strength training throughout the duration of the study.

### Strength Training

The subjects assigned to the strength training group visited the laboratory for supervised strength training twice per week for ten weeks. Each training session involved conventional barbell deadlifts with the feet placed roughly shoulder width apart. A minimum of 48 hours of rest was required between training sessions. The subjects received personal instruction and verbal feedback regarding their exercise technique throughout the entire study. Since the barbell deadlift has the potential to result in injuries to the musculature of the lower back in untrained subjects, maximal strength testing of this exercise was not performed. Instead, we sought to determine the heaviest external load that allowed each subject to perform five sets of five repetitions with correct technique. To accomplish this, the subjects began their first training session with an external load of 61.4 kg, and weight was added based on the subject's ability to perform a set. Each training session began with two warm-up sets of five repetitions. Three minutes of rest was allotted between each set. As a means of progressive overload, 0.45–2.2 kg was added to the barbell for each training session. In the event that five repetitions within a set could not be completed, or if the exercise technique became compromised because of fatigue, 0.45–2.2 kg was removed from the barbell. If the subjects were unable to complete five repetitions for each of the five sets, a sixth set was allowed so that 25 repetitions could be performed. All of the subjects in the training group performed a total of 25 repetitions for each of the 20 training sessions. The mean ± SD external load used to perform the 25 repetitions during the final training session was 124.6±20.3 kg. For the subjects in the training group, the post-test was scheduled a minimum of 72 hours following the final training session.

### Isometric Force Testing

On a separate day 24–48 hours prior to the pre-test, the subjects were familiarized with the data collection procedures and became comfortable performing single-joint MVCs of the knee extensors, as well as steady increases and decreases in submaximal force. Upon arrival to the Muscular Assessment Laboratory, the subjects were seated in a modified knee extension chair that allowed for force testing of the isolated knee joint. The subjects were restrained to the chair via straps that were secured around the chest, abdomen, and hips. A Velcro cuff was secured around the right ankle joint, which was attached to a calibrated tension/compression load cell (Model SSM-AJ-500; Interface, Scottsdale, AZ) to allow for the measurement of isometric force. All maximal and submaximal force testing occurred at a knee joint angle of 60° below the horizontal plane. This joint angle was similar to that used during the initiation of each repetition of the barbell deadlift exercise for the majority of the subjects, and this was verified with a goniometer. Following a brief submaximal warm-up period, the subjects performed two, three-second MVCs separated by three minutes. The highest value from the two trials was chosen as the MVC, and was used to standardize the submaximal testing among the subjects. Following the determination of the MVC, the subjects performed a trapezoidal isometric contraction in accordance with a visual template on a computer monitor. The subjects increased isometric force from 0–50% MVC in five seconds (10%/second), held 50% constant for ten seconds, and decreased isometric force from 50–0% MVC in five seconds (10%/second). The subjects were instructed to maintain their force output as close as possible to the target force. Force steadiness was defined as the coefficient of variation ([SD/mean] ×100) over the entire ten second constant-force portion of the contraction. During post-testing, the subjects performed trapezoidal isometric contractions at absolute force levels corresponding to 50% of the pre-test MVC, as well as that for the post-test value. For example, if a subject in the strength training group demonstrated MVCs of 500 and 700 N for the pre-test and post-test, the constant-force levels corresponded to 250 and 350 N, respectively. A three minute rest period was provided between all contractions. Using the procedures described by Weir [24], a test-retest reliability analysis for our laboratory's MVC force values in eleven subjects demonstrated an intraclass correlation coefficient (model 3,1) of 0.949, with no significant difference between the trials (*p* = 0.867). For each subject, the pre- and post-test isometric force assessment sessions occurred at approximately the same time of day (±1 hour).

### Surface EMG Signal Recording

Surface EMG signals were recorded from the vastus lateralis and rectus femoris during each of the submaximal contractions with a Bagnoli 16-channel Desktop system (Delsys, Inc., Boston, MA). Prior to detecting EMG signals, the skin over the muscles and patella was shaved and cleansed with rubbing alcohol. Oil, debris, and dead skin cells were also removed with hypo-allergenic tape. The sensors were placed over the muscles in accordance with the recommendations described Zaheer et al. [25]. A reference electrode was placed over the patella. The signals were detected with two separate surface array EMG sensors (Delsys, Inc., Boston, MA) that each consist of five pin electrodes [17]. Four of the five electrodes are arranged in a square, with the fifth electrode in the center of the square and at a fixed distance of 3.6 mm from all other electrodes. Pairwise subtraction of the five electrodes was used to derive four single differential EMG channels for each muscle. These signals were differentially amplified, filtered with a bandwidth of 20 Hz to 1,750 Hz, and sampled at 20 kHz. Surface EMG signal quality (i.e., signal-to-noise ratio >3.0, baseline noise value ≤2.0 µV root-mean-square, line interference <1.0) was verified for a 20% MVC assessment prior to data acquisition. The mean ± SD pre-test skinfold thickness values for the vastus lateralis and rectus femoris were 14.4±7.5 and 18.5±9.1 mm, respectively, and these values did not change (*p*>.05).

### Surface EMG Signal Decomposition

**Figure 1:**
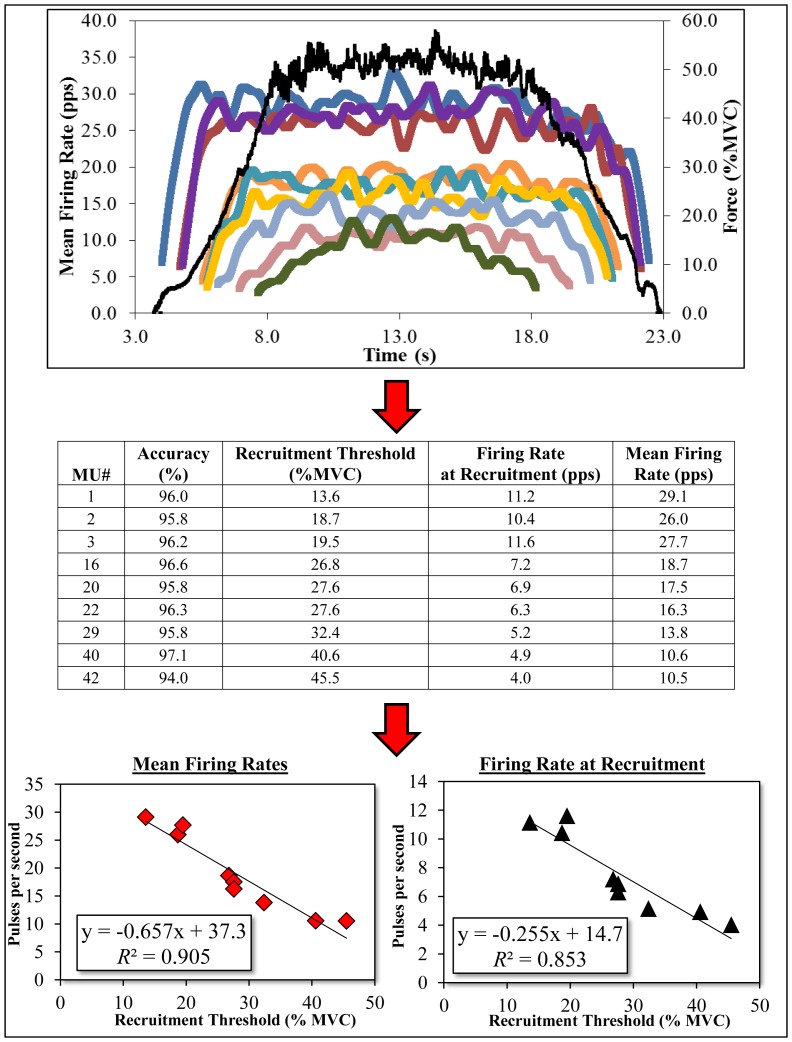
Example time-varying motor unit firing rate curves for the vastus lateralis of one subject during the pre-test. For this contraction, the algorithm was able to decompose 42 motor units with greater than 92.0% accuracy, but nine have been displayed here for visual clarity. The table and graphs below the mean firing rate plot display the accuracy level, recruitment threshold, mean firing rate, and firing rate at recruitment for each motor unit.

The eight separate filtered EMG signals from the two muscles served as the input to the Precision Decomposition III algorithm. For further information concerning the technical aspects of this algorithm, the reader is directed to the work of Nawab et al. (2010). The surface EMG signals were decomposed into their constituent motor unit action potential trains. These trains were then used to calculate a time-varying firing rate curve for each detected motor unit. All firing rate curves were smoothed with a one-second Hanning filter, and selected from the six-second middle portion (i.e., seconds 10–16) of the constant-force contraction. The mean number of pulses per second (pps) for each six-second motor unit firing rate curve was calculated. High threshold motor units that were recruited or derecruited during the constant-force portion of the protocol and therefore not active throughout the entire six-second portion of the firing rate curve were not considered for data analysis. The firing rate at recruitment was estimated from the inverse of the mean of the first three interpulse intervals [13]. Each motor unit's recruitment threshold was calculated as the relative force level (% MVC) when the first firing occurred. Fig. 1 displays example mean firing rate curves and their associated statistics for nine vastus lateralis motor units. The horizontal axis corresponds to time in seconds, whereas the left and right vertical axes display the firing rate (pulses per second) and the percentage of the MVC, respectively. The black line corresponds to this subject's force output. Each of the nine colored lines represents the time-varying firing rate of an individual motor unit. Fig. 1 also displays examples of the accuracy, recruitment threshold, firing rate at recruitment, and mean firing rate for each motor unit. The bottom two scatterplots exemplify the relationship between mean firing rate versus recruitment threshold (left), as well as firing rate at recruitment versus recruitment threshold (right). The data and analyses shown in Fig. 1 demonstrate how all of the statistical outcomes for this study were determined.

### Motor Unit Decomposition Accuracy

Once all of the signals were successfully decomposed, the Decompose-Synthesize-Decompose-Compare test was used to determine the accuracy of each motor unit [13]. Motor units with accuracy levels less than 92.0% were removed from further statistical analyses. In the majority of the cases, the accuracy levels of the detected motor units were 94.0% or greater (mean ± SD  = 95.3±2.2). Two addition steps were taken to increase the validity of our procedures. First, contractions that yielded less than six motor units were removed from consideration. Furthermore, contractions that yielded motor units with a recruitment threshold range of less than 10.0% were also removed from consideration. For a few contractions, very low threshold motor units were detected just prior to the onset of measureable force (i.e., recruitment thresholds at 0% MVC). These motor units were not considered for further statistical analysis. Table 1 displays individual contraction data for the number of motor units that were successfully analyzed.

**Table 1:** Individual contraction data for the number of motor units decomposed with greater than 92.0% accuracy.

Pre-test				Post-test; pre-test force				Post-test; new force			
Training		Control		Training		Control		Training		Control	
ID	#VL/#RF	ID	#VL/#RF	ID	#VL/#RF	ID	#VL/#RF	ID	#VL/#RF	ID	#VL/#RF
ST001	22/28	C001	25/24	ST001	25/30	C001	29/28	ST001	24/29	C001	21/39
ST002	20/31	C002	24/37	ST002	31/32	C002	28/29	ST002	17/36	C002	28/30
ST003	28/33	C003	25/14	ST003	10/16	C003	24/36	ST003	15/41	C003	29/35
ST004	20/28	C004	41/35	ST004	31/23	C004	35/21	ST004	28/21	C004	36/18
ST005	26/28	C005	29/26	ST005	18/30	C005	33/11	ST005	33/38	C005	31/7
ST006	31/24	C006	16/29	ST006	33/31	C006	32/17	ST006	32/30	C006	16/25
ST007	23/12	C007	21/41	ST007	22/20	C007	32/31	ST007	13/32	C007	34/32
ST008	23/33	C008	22/14	ST008	24/28	C008	36/25	ST008	23/36	C008	47/24
ST009	32/30	C009	25/25	ST009	29/25	C009	41/33	ST009	24/31	C009	37/29
ST010	26/16			ST010	27/15			ST010	24/10		
ST011	25/23			ST011	26/41			ST011	28/38		
ST012	19/35			ST012	18/32			ST012	22/37		
ST013	23/24			ST013	23/21			ST013	28/22		
ST014	31/30			ST014	22/26			ST014	21/34		
ST015	25/42			ST015	26/47			ST015	24/29		
Sum	374/417	Sum	228/245	Sum	365/417	Sum	290/231	Sum	356/464	Sum	279/239
Mean	25/28	Mean	25/27	Mean	24/28	Mean	32/26	Mean	24/31	Mean	31/27

VL  =  vastus lateralis; RF  =  rectus femoris; ST  =  strength training; C  =  control.

### Statistical Analyses

Descriptive statistics for the mean firing rates and firing rates at recruitment for the vastus lateralis and rectus femoris have been displayed in Table 2. For both muscles, linear regression analyses were performed on the mean firing rate and firing rate at recruitment versus the recruitment threshold to determine the slope coefficients (pps/% MVC) and y-intercepts (pps) of each relationship. Thus, there were eight separate motor unit dependent variables examined in this investigation. A two-way mixed-factorial (time [pre-test, post-test] × group [training, control]) analysis of variance (ANOVA) was used to examine mean differences for the MVC values. Nine separate two-way mixed factorial (force [pre-test 50% MVC, post-test original 50% MVC, post-test new 50% MVC] × group [training, control]) ANOVAs were used to examine force steadiness, as well as the linear slope coefficients and y-intercepts for the motor unit regression variables. When appropriate, follow-up analyses included repeated measures ANOVAs, dependent samples *t*-tests, and Bonferroni post-hoc comparisons. In addition, partial eta squared (ή^2^) and Cohen's *d* statistics were examined when necessary. According to Stevens [26], partial eta squared values of .01, .06, and.14 correspond to small, moderate, and large effect sizes, respectively. Cohen [27] described *d* values of 0.20, 0.50, and 0.80 as corresponding to small, moderate, and large effect sizes, respectively. An alpha level of .05 was used to determine statistical significance for all analyses. The statistical analyses were performed using SPSS software (version 21.0, SPSS Inc., Chicago, IL).

**Table 2:** Descriptive statistics for the mean firing rates and firing rates at recruitment for the vastus lateralis and rectus femoris.

	Strength Training Group (*n* = 15)			Control Group (*n* = 9)		
	Pre-test	Post-test; pre-test force	Post-test; new force	Pre-test	Post-test; pre-test force	Post-test; new force
**Vastus Lateralis Mean Firing Rates**						
Mean	20.5	20.0	18.9	20.5	19.3	18.9
SD	4.4	5.7	5.3	4.8	4.8	5.3
Range	9.3 to 36.2	9.7 to 32.5	8.7 to 30.9	9.1 to 31.7	6.2 to 33.4	8.7 to 30.9
**Rectus Femoris Mean Firing Rates**						
Mean	18.6	19.2	18.0	19.8	18.1	17.7
SD	4.2	4.1	4.3	3.8	3.8	4.1
Range	8.5 to 28.1	9.1 to 31.2	8.2 to 29.6	10.2 to 29.8	7.6 to 27.7	7.3 to 27.5
**Vastus Lateralis Firing Rates at Recruitment**						
Mean	7.9	7.3	7.2	7.8	7.1	7.2
SD	2.0	2.5	2.4	2.1	2.3	2.4
Range	3.6 to 15.1	2.2 to 13.8	2.4 to 13.8	3.8 to 13.0	1.7 to 15.4	2.4 to 13.8
**Rectus Femoris Firing Rates at Recruitment**						
Mean	6.6	6.9	6.8	7.2	6.3	6.4
SD	1.7	2.1	2.0	2.0	1.8	1.8
Range	2.7 to 11.8	2.5 to 15.0	2.1 to 12.4	2.7 to 12.8	2.3 to 12.5	1.9 to 10.8

Statistical analyses have not been performed on these data due to the fact that a motor unit's firing rate is dependent on its recruitment threshold. Post-testing involved assessing the same absolute force level examined during the pre-test, as well as 50% of the new MVC value. Note the similar firing rates for the two muscles. All values are in units of pulses per second.

## Results

### MVC Force and Force Steadiness

**Figure 2:**
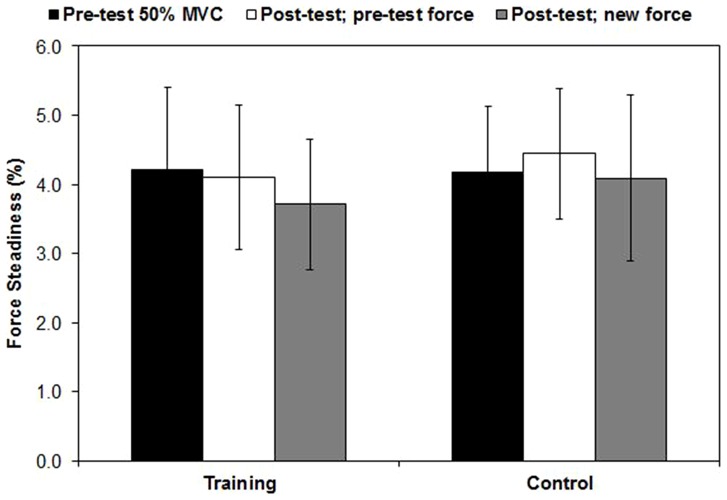
Mean ± SD force steadiness values. Force steadiness was defined as the coefficient of variation over the ten second constant-force portion of the contraction. During post-testing, the subjects performed trapezoidal isometric contractions at absolute force levels corresponding to 50% of the pre-test MVC, as well as that for the new post-test value.

For the MVC force two-way mixed factorial ANOVA, there was a two-way interaction (*p* = .011, ή^2^ = .262). The results from two separate dependent samples *t*-tests indicated that the MVC values increased for the training group (*p* = .002, Cohen's *d* = .70), but not the control group (*p* = .992, Cohen's *d*<.01). For force steadiness, the results from the two-way mixed factorial ANOVA indicated that there was no interaction (*p* = .580, ή^2^ = .024) and no main effect for force level (*p* = .096, ή^2^ = .101) or group (*p* = .434, ή^2^ = .028 [Fig. 2]).

### Mean Firing Rate versus Recruitment Threshold Relationship for the Vastus Lateralis

For the linear slope coefficients, the results from the two-way mixed factorial ANOVA indicated that there was no interaction (*p* = .132, ή^2^ = .088), and no main effect for force level (*p* = .091, ή^2^ = .103) or group (*p* = .397, ή^2^ = .033). For the y-intercepts, the results indicated that there was no interaction (*p* = .627, ή^2^ = .021) and no main effect for group (*p* = .797, ή^2^ = .003). There was, however, a main effect for force level (*p* = .032, ή^2^ = .145). The results from the Bonferroni marginal mean pairwise comparison indicated that when collapsed across group, the y-intercepts for post-test assessment involving 50% of the pre-test MVC (26.0 pps) were significantly less than those for the pre-test (29.8 pps). The 95% CI for this mean difference was .90 to 6.64 pps (*p* = .008 [Table 3]).

**Table 3:** Means, SDs, 95% confidence intervals (CIs) and ranges for the linear slope coefficients and y-intercepts for the relationships between motor unit mean firing rate versus recruitment threshold for the vastus lateralis and rectus femoris.

	Strength Training Group (*n* = 15)			Control Group (*n* = 9)		
	Pre-test	Post-test; pre-test force	Post-test; new force	Pre-test	Post-test; pre-test force	Post-test; new force
**Vastus Lateralis Linear Slope Coefficient of Mean Firing Rate versus Recruitment Threshold (pps/% MVC)**						
Mean ± SD	−0.45±0.18	−0.45±0.17	−0.48±0.28	−0.60±0.19	−0.40±0.31	−0.58±0.23
95% CI	−0.54 to −0.35	−0.55 to −0.36	−0.63 to −0.33	−0.74 to −0.45	−0.63 to −0.17	−0.75 to −0.40
Range	−0.82 to −0.23	−0.77 to −0.19	−1.36 to −0.25	−0.83 to −0.34	−1.15 to −0.20	−0.90 to −0.19
**Rectus Femoris Linear Slope Coefficient of Mean Firing Rate versus Recruitment Threshold (pps/% MVC)**						
Mean±SD	−0.46±0.21	−0.55±0.27	−0.54±0.18	−0.47±0.23	−0.38±0.23	−0.47±0.26
95% CI	−0.58 to −0.35	−0.70 to −0.40	−0.63 to −0.44	−0.64 to −0.29	−0.56 to −0.21	−0.67 to −0.27
Range	−0.88 to −0.22	−1.25 to −0.20	−0.86 to −0.24	−1.03 to −0.28	−0.71 to −0.19	−0.81 to −0.09
**Vastus Lateralis Y-Intercept of Mean Firing Rate versus Recruitment Threshold (pps)**						
Mean±SD	28.9±4.4	25.5±4.1	28.6±8.5	30.6±6.6	26.6±9.0	27.7±9.4
95% CI	26.5 to 31.4	23.2 to 27.7	23.9 to 33.3	25.6 to 35.7	19.7 to 33.5	20.5 to 35.0
Range	22.3 to 38.4	17.3 to 31.0	17.1 to 52.1	24.4 to 42.9	17.1 to 46.7	13.9 to 47.1
**Rectus Femoris Y-Intercept of Mean Firing Rate versus Recruitment Threshold (pps)**						
Mean±SD	32.5±7.2	34.4±8.7	34.5±7.0	31.5±8.3	26.2±7.6	30.0±9.2
95% CI	28.5 to 36.5	29.5 to 39.2	30.7 to 38.4	25.1 to 37.9	20.4 to 32.0	22.9 to 37.1
Range	24.3 to 48.7	24.3 to 53.5	20.7 to 49.0	18.2 to 48.5	16.0 to 36.7	20.0 to 44.3

Post-testing involved assessing the same absolute force level examined during the pre-test, as well as 50% of the new MVC value. The results from the two-way mixed factorial analyses of variance indicated that there were no significant changes as a result of strength training.

### Mean Firing Rate versus Recruitment Threshold Relationship for the Rectus Femoris

For the linear slope coefficients, the results from the two-way mixed factorial ANOVA indicated that there was no interaction (*p* = .202, ή^2^ = .071), and no main effect for force level (*p* = .657, ή^2^ = .109) or group (*p* = .337, ή^2^ = .042). For the y-intercepts, there was no interaction (*p* = .079, ή^2^ = .109), and no main effect for force level (*p* = .384, ή^2^ = .043) or group (*p* = .121, ή^2^ = .106 [Table 3]).

### Firing Rate at Recruitment versus Recruitment Threshold Relationship for the Vastus Lateralis

For the linear slope coefficients, the results from the two-way mixed factorial ANOVA indicated that there was no interaction (*p* = .827, ή^2^ = .009), and no main effect for force level (*p* = .153, ή^2^ = .082) or group (*p* = .491, ή^2^ = .022). For the y-intercepts, the results indicated that there was no interaction (*p* = .665, ή^2^ = .018) and no main effect for group (*p* = .963, ή^2^<.001). There was, however, a main effect for force level (*p* = .041, ή^2^ = .135). The results from the Bonferroni marginal mean pairwise comparison indicated that when collapsed across group, the y-intercepts for post-test assessment involving 50% of the pre-test MVC (9.6 pps) were significantly less than those for the pre-test (11.4 pps). The 95% CI for this mean difference was .22 to 3.36 pps (*p* = .022 [Table 4]).

**Table 4:** Means, SDs, 95% confidence intervals (CIs), and ranges for the linear slope coefficients and y-intercepts for the relationships between motor unit firing rate at recruitment versus recruitment threshold for the vastus lateralis and rectus femoris.

	Strength Training Group (*n* = 15)			Control Group (*n* = 9)		
	Pre-test	Post-test; pre-test force	Post-test; new force	Pre-test	Post-test; pre-test force	Post-test; new force
**Vastus Lateralis Linear Slope Coefficient of Firing Rate at Recruitment versus Recruitment Threshold (pps/% MVC)**						
Mean±SD	−0.18±0.08	−0.14±0.12	−0.18±0.15	−0.22±0.17	−0.14±0.15	−0.23±0.19
95% CI	−0.23 to −0.14	−0.21 to −0.07	−0.27 to −0.10	−0.35 to −0.09	−0.26 to −0.03	−0.37 to −0.08
Range	−0.35 to −0.08	−0.36 to 0.03	−0.54 to 0.11	−0.57 to −0.02	−0.52 to 0.02	−0.64 to −0.02
**Rectus Femoris Linear Slope Coefficient of Firing Rate at Recruitment versus Recruitment Threshold (pps/% MVC)**						
Mean±SD	−0.13±0.12	−0.16±0.17	−0.16±0.15	−0.07±0.20	−0.03±0.11	−0.02±0.21
95% CI	−0.20 to −0.07	−0.25 to −0.06	−0.24 to −0.07	−0.22 to −0.09	−0.11 to 0.06	−0.18 to 0.14
Range	−0.35 to 0.04	−0.54 to 0.07	−0.39 to 0.12	−0.47 to 0.12	−0.17 to 0.17	−0.32 to 0.43
**Vastus Lateralis Y-Intercept of Firing Rate at Recruitment versus Recruitment Threshold (pps)**						
Mean±SD	11.3±1.7	9.3±2.1	11.0±4.2	11.5±3.4	9.9±4.5	10.4±4.4
95% CI	10.4 to 12.3	8.1 to 10.5	8.7 to 13.3	8.8 to 14.1	6.4 to 13.4	7.0 to 13.8
Range	8.7 to 13.9	6.0 to 12.8	4.5 to 20.9	6.8 to 16.6	5.8 to 20.3	4.6 to 19.6
**Rectus Femoris Y-Intercept of Firing Rate at Recruitment versus Recruitment Threshold (pps)**						
Mean±SD	11.0±4.5	11.7±5.3	12.3±4.9	9.2±6.6	7.2±3.1	8.2±4.2
95% CI	8.5 to 13.4	8.8 to 14.7	9.6 to 15.0	4.1 to 14.2	4.8 to 9.5	5.0 to 11.4
Range	5.4 to 19.1	5.2 to 22.5	3.7 to 21.8	2.1 to 21.2	2.7 to 12.3	8.1 to 17.7

Post-testing involved assessing the same absolute force level examined during the pre-test, as well as 50% of the new MVC value. The results from the two-way mixed factorial analyses of variance indicated that there were no significant changes as a result of strength training.

### Firing Rate at Recruitment versus Recruitment Threshold Relationship for the Rectus Femoris

For the linear slope coefficients, the results from the two-way mixed factorial ANOVA indicated that there was no interaction (*p* = .544, ή^2^ = .027), and no main effect for force level (*p* = .929, ή^2^ = .003). There was, however, a main effect for group (*p* = .046, ή^2^ = .170). The results from the Bonferroni marginal mean pairwise comparison indicated that when collapsed across force level, the linear slope coefficients for the subjects in the training group were significantly less than those for the control group (−0.148 versus −0.038 pps/% MVC). The 95% CI for this mean difference was −0.217 to 0.002 pps/% MVC (*p* = .046). For the y-intercepts, there was no interaction (*p* = .345, ή^2^ = .047), and no main effect for force level (*p* = .705, ή^2^ = .016) or group (*p* = .054, ή^2^ = .158 [Table 4]).

## Discussion

Previous investigations have demonstrated that the increase in force production as a result of strength training is due, at least in part, to neural factors [28], [29], one of which may be an alteration in the firing rates of motor units. Theoretically, improvements in the ability to produce force as a result of training may affect motor unit behavior in two ways: 1) an increase in firing rates at higher absolute force levels [7], and/or 2) a decrease in firing rates at absolute force levels corresponding to pre-training relative percentages of the MVC [22]. The results of this study showed that a ten week strength training program which improved MVC force had no influence on mean motor unit firing rates for the vastus lateralis and rectus femoris. In addition, this investigation was the first to examine changes in the firing rates at recruitment as a result of strength training, and we conclude that this parameter was also unaffected. Our findings revealed no improvements in force steadiness (Fig. 2), which is in agreement with a recent investigation [5], but not with those that have studied elderly subjects [30], [31] or clinical populations [32]. This investigation was also the first to fully describe the submaximal firing rates of the rectus femoris using surface EMG signal decomposition, and our results demonstrated similar statistics compared with those for the vastus lateralis, as well as the vastus medialis [18]. Technological advances in the capacity to accurately decompose surface EMG signals allowed us to meticulously quantify the recruitment thresholds and firing rates of a large sample of motor units from 24 subjects and two muscles, thereby giving us great confidence in the validity of our conclusions.

Our findings are largely in agreement with previous studies that used comparable methods [4], [5], [8], but are in disagreement with the results reported by Vila-Cha and colleagues [9], who compared training adaptations to strength versus endurance training. Although the discrepancies among studies that have used randomized control designs are vast, both the exercise selection and the specificity of the forces/external loads applied during training may be critical. One of the unique aspects of the present study was the fact that the subjects were taught how to perform a free-weight, barbell exercise. In contrast to each of the previous investigations that have used exercise machines, the barbell deadlift requires significant balance, proprioception, and coordination through a relatively large range of motion [21], [33]. As a result, the movement pattern for the deadlift could be considered more functional and relevant to activities of daily living. This study expands on the previous investigations that have examined changes in motor unit firing rates following strength training, as our results show that exercise selection may not be a critical aspect as it relates to changes for an individual muscle during a single-joint task even when MVC values improve. Although the training program utilized in this study did improve isometric force production, it also seems reasonable to speculate that motor unit adaptations may have been specific to the movement pattern associated with the deadlift exercise. This phenomenon was first illustrated by Rutherford et al. [34], who demonstrated nearly a 200% increase in the external loads lifted by subjects, but only a 3–20% increase in isometric force. In fact, the issue of testing versus training specificity explains the discrepant findings reported by a variety of authors [4], [7], [8], [35], with changes in firing rates typically demonstrated when subjects are asked to perform the same task throughout the study. As the noninvasive assessment of motor unit firing rates is currently limited to isometric testing modes [17], the ability to draw conclusions regarding adaptations to dynamic exercise remains somewhat limited.

When examining many of the previous studies that have investigated firing rate adaptations following improvements in force via strength training [4]–[10], it becomes clear that there are two additional considerations that may explain the inconsistencies among findings. First, the testing schedule relative to improvements in maximal force seems important. Patten et al. [7] studied abductor digiti minimi motor unit firing rates in six young and six elderly adults before and following six weeks of strength training. It was reported that firing rates for the trained hand increased by 24% only 48 hours after the initial experimental session. This was noted in spite of a minor increase in MVC force. Interestingly, the firing rates decreased thereafter, and similar values were shown among the pre-test and two and six week assessments. This finding was replicated for the vastus lateralis in an investigation by Kamen and Knight [10], who reported a 19% increase in firing rates following the initial training session. Thus, the results from these two studies [7], [10] indicate that increases in motor unit firing rates may explain the improvement in maximal force during the initial period of exercise and/or repeated testing, but their importance diminishes as training progresses for several weeks. The second methodological factor that must be considered is the force level used during testing, as dissimilarities in conclusions could be explained by whether assessments are maximal versus submaximal. The previously mentioned investigations by Kamen and Knight [10] and Patten et al. [7] examined maximal firing rates. With the exception of one study [9], each of the previous experiments that noted improvements in MVC force but assessed motor unit behavior during submaximal contractions found no change in firing rates [4]–[6], [8]. It is reasonable to hypothesize that strength training plays an important role in motor unit adaptation for maximal force contractions and/or improving the rate of force development [36], and that these effects are not measureable during submaximal contractions. However, no previous study has examined firing rates during submaximal contractions following a period of less than one week of strength training. Overall, we speculate that if strength training does influence the regression coefficients associated with mean and initial firing rates, the effects may only be evident during maximal contractions following very short-term training [7], [35], [36]. In agreement with previous cross-sectional data [11], our findings do not support the notion that exposure to strength training modifies the control of motor units during submaximal contractions.

The values that we have displayed in Tables 2–4 are generally in agreement with those shown in our previous work concerning motor unit fatigue [18], the experiment performed by Beck et al. [4], and by others that have studied 50% MVC force levels with surface EMG signal decomposition [13], [14]. As calculated from Table 2, the average of the mean firing rates during the 50% MVC assessment was 19.7 pps for the vastus lateralis. The corresponding value for the rectus femoris was 18.6 pps, suggesting that these two muscles display comparable mean firing rate characteristics during submaximal extension at the knee joint. When examined on an individual subject basis for the vastus lateralis, the highest and lowest mean firing rate was 36.2 and 6.2 pps, respectively. The corresponding values for the rectus femoris were 31.2 and 7.3 pps. Furthermore, as shown in Table 3 (and exemplified in Fig. 1), all of the linear slope coefficients for the mean firing rate versus recruitment threshold relationships were negative, with none of the 95% CIs including zero. The inverse relationship between the mean firing rates of motor units and their recruitment thresholds is consistent with the “onion skin” phenomenon, which was described in detail by De Luca and Hostage [14] as an evolutionary means of optimizing the combination of force magnitude over time. Data concerning the firing rate at recruitment have been displayed in both Table 2 and Table 3. When both muscles are combined, the average firing rate at recruitment value was 7.1 pps, with peak values of 15.4 and 15.0 for the vastus lateralis and rectus femoris, respectively. As shown in Table 4, each of the mean linear slope coefficients for the firing rate at recruitment versus recruitment threshold relationships were negative, which is consistent with the work of Tanji and Kato [37] and De Luca and Contessa [13]. However, we should point out that in some cases, weakly positive relationships were demonstrated, or no relationship was shown at all (*r* = ∼0.0). Specifically, four and thirteen contractions for the vastus lateralis and rectus femoris, respectively, demonstrated positive relationships. This was more apparent for the subjects in the control group for the rectus femoris, leading to a main effect and y-intercepts closer to 0 pps. As pointed out by De Luca and Contessa [13], a few studies that examined initial firing rates have noted positive relationships when regressed against recruitment threshold [38]–[40], but it was noted that differences in the algorithms used to detect the early firings of motor units may explain the discrepancy. Since the rate of force increase during the isometric contractions was the same for each test (10%/second), the exact reasoning for this is unclear, although we speculate that it could be related to synaptic noise [41], or the means of estimating initial firing rates from only a few interpulse intervals. Alternatively, it is possible that a 92% accuracy cutoff was not rigorous enough, and some of the initial firings should not have been considered for data analysis. Nonetheless, the values displayed in Tables 1–3 can be summarized as follows: 1) the vastus lateralis and rectus femoris exhibit similar firing rates during 50% MVC isometric force testing, 2) the mean firing rates of both muscles are inversely related with their recruitment thresholds, 3) the firing rates at recruitment are also inversely related with their recruitment thresholds, but the response is not as consistent or linear as that demonstrated for mean firing rates, and 4) none of these statistics are affected by strength training regardless of whether qualitative examination of the data (Table 2) or regression-based group mean statistical analyses (Tables 3 and 4) are used.

Our experimental approach requires discussion of a final methodological consideration that, to our knowledge, has not been contemplated by previous authors. This investigation was unique due to the fact that the post-test involved two submaximal contractions. The first assessment required the subjects to perform a trapezoidal isometric contraction corresponding to 50% of the new MVC. In contrast, the second contraction was performed at an absolute force level corresponding to 50% of the pre-test value. Thus, for subjects that demonstrated increased MVC values due to the training program, we were able to examine the firing rates corresponding to both force levels. In contrast, those subjects assigned to the control group performed two contractions at relatively similar isometric force levels. We believe that this is an important consideration because if only relative force levels are studied for both the pre-test and post-test (e.g., 60% MVC), but the MVC values change, one could rightfully contend that the differences in firing rates are simply a reflection of the dissimilar absolute force levels examined, and not an adaptation from the exercise stimulus. By studying two force levels, we are able to confirm that the acquisition of strength following ten weeks of training had no influence on the firing rates of motor units, and this finding is robust for both relative and absolute isometric force levels.

## Conclusion

Ten weeks of barbell deadlift training did not affect the mean firing rates, as well as the firing rates at recruitment, of motor units for the vastus lateralis and rectus femoris. These findings were in spite of improvements in the knee extension MVC values. Our results are in close agreement with previous investigations that have reported no change in the firing rates of motor units during submaximal contractions [4]–[6], [8], but are in opposition to those that examined maximal contractions [7], [10]. Furthermore, the training program did not affect force steadiness, which is in agreement with a previous investigation that had subjects perform leg press and extension exercises [5]. Although the reason for the lack of improvement in force steadiness is unclear, we speculate that young, healthy subjects that are adequately familiarized with isometric testing have little room for improvement, particularly for those that demonstrate a coefficient of variation less than 4.0% during pre-testing. It should be noted that we studied the linear slope coefficients and y-intercepts of mean firing rate and firing rate at recruitment and versus recruitment threshold relationships, and other methods of quantifying the behavior of motor units (e.g., synchronization, common drive, variability of interpulse intervals) were not considered. It is possible that had other variables been examined, dissimilar findings would have been discovered, although we speculate that this is unlikely [6], [11].
